# Controlling Redox Status for Stem Cell Survival, Expansion, and Differentiation

**DOI:** 10.1155/2015/105135

**Published:** 2015-07-27

**Authors:** Sébastien Sart, Liqing Song, Yan Li

**Affiliations:** ^1^Hydrodynamics Laboratory, CNRS UMR 7646, Ecole Polytechnique, 91120 Palaiseau, France; ^2^Department of Chemical and Biomedical Engineering, FAMU-FSU College of Engineering, Florida State University, 2525 Pottsdamer Street, Tallahassee, FL 32310, USA

## Abstract

Reactive oxygen species (ROS) have long been considered as pathological agents inducing apoptosis under adverse culture conditions. However, recent findings have challenged this dogma and physiological levels of ROS are now considered as secondary messengers, mediating numerous cellular functions in stem cells. Stem cells represent important tools for tissue engineering, drug screening, and disease modeling. However, the safe use of stem cells for clinical applications still requires culture improvements to obtain functional cells. With the examples of mesenchymal stem cells (MSCs) and pluripotent stem cells (PSCs), this review investigates the roles of ROS in the maintenance of self-renewal, proliferation, and differentiation of stem cells. In addition, this work highlights that the tight control of stem cell microenvironment, including cell organization, and metabolic and mechanical environments, may be an effective approach to regulate endogenous ROS generation. Taken together, this paper indicates the need for better quantification of ROS towards the accurate control of stem cell fate.

## 1. Introduction

Mesenchymal stem cells (MSCs) and pluripotent stem cells (PSCs), including embryonic stem cells (ESCs) and induced pluripotent stem cells (iPSCs), have emerged as important tools for drug screening, disease modeling, and tissue engineering [1, 2]. MSCs are progenitors of connective tissues, bearing differentiation potential along osteoblasts, chondrocytes, and adipocytes [3]. MSCs are now evaluated in more than 400 clinical trials due to their differentiation potential and especially their trophic activities (i.e., the secretion of antiapoptotic, anti-inflammatory, and antiscarring factors), which constitute their major therapeutic effects* in vivo* [1]. Different from MSCs, ESCs are derived from inner mass of the blastocyst and iPSCs are obtained by reprogramming somatic cells to ESC-like pluripotent state by overexpression of the pluripotent genes [4]. Both cell populations have differentiation potential for a large spectrum of somatic cell types, mimicking the embryonic development. However, there is still a limited control of lineage-specific differentiation, which impedes the high promise of PSCs for the treatment of incurable diseases [5]. For MSCs, the limited efficacy of MSCs* in vivo* also indicates the need to improve their therapeutic functions* in vitro* prior to transplantation [6].

Once injected into damaged tissues, stem cells are exposed to acute ischemia and oxygen deprivation, which lead to the production of highly oxidizing compounds, known as reactive oxygen species (ROS). Excessive ROS would result in the apoptosis of the transplanted cells [7]. Similarly, exposure of stem cells to extreme culture conditions* in vitro* (such as starvation, metabolic alterations, and exposure to toxic molecules) also leads to the apoptosis mediated by ROS [8, 9]. Thus, ROS has been recognized as pathological metabolic agents that reduce stem cell functions. However, recent studies have challenged this dogma by demonstrating the positive effects of physiological ROS for the regulation of stem cell fate decision. For instance, hypoxia results in mild levels of ROS (e.g., 1.8-fold of normal level), which are actively involved in the regulation of proliferation and differentiation of MSCs and PSCs [10, 11]. Moreover, the metabolic shift observed during stem cell commitment leads to the increased levels of ROS which are intrinsically linked with the differentiation stage of stem cells [12]. Hence, it is becoming clear that physiological levels of ROS play a role of secondary messengers in the regulation of stem cell fate. As a consequence, the control of ROS generation could lead to efficient stem cell expansion and differentiation.

This review investigates recent advances in the understanding of ROS generation and the mechanisms to sustain the redox equilibrium in MSCs and PSCs. In addition, this paper underlines how ROS positively or negatively interferes with the signaling pathways that regulate stem cell survival, proliferation and differentiation. Novel strategies for the tight regulation of stem cell microenvironment which enables the modulation of cellular redox status to control stem cell fate are also discussed.

## 2. ROS Generation and Scavenging in Stem Cells

Stem cell physiology and metabolism are tightly regulated by oxidation-reduction events that mainly occur during respiratory chain. To maintain the redox equilibrium, the oxidative status in stem cells is regulated by the controlled balance of ROS production and scavenging, through the generation of endogenous antioxidants. Therefore, understanding the cellular redox state is important to modulate stem cell survival, expansion, and differentiation.

### 2.1. ROS Generation in Stem Cells

**Figure 1:**
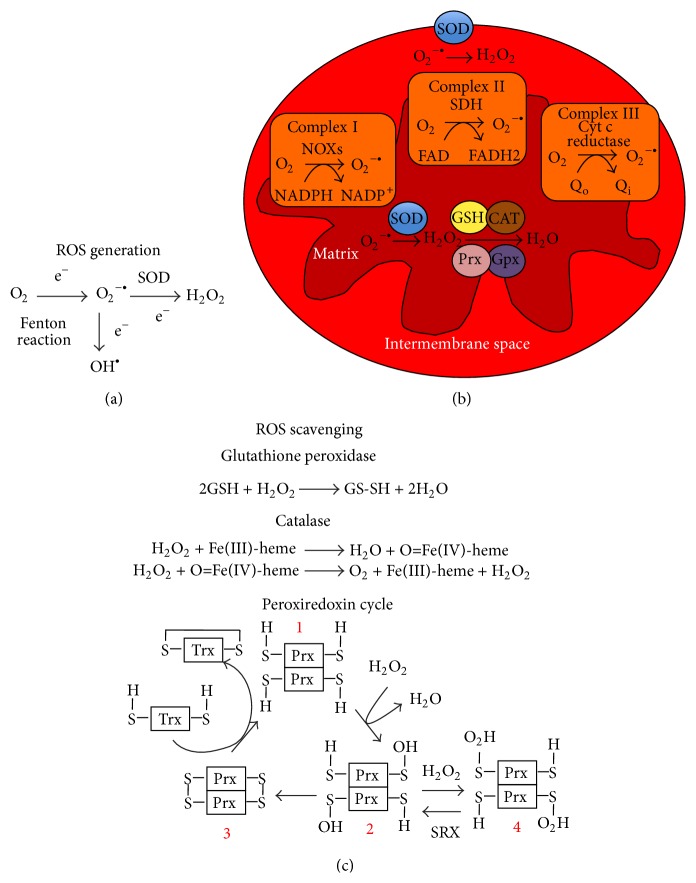
ROS generation and scavenging in stem cells. (a) ROS generation. ROS generation is initiated with the electron transfer to molecular O_2_, leading to the formation superoxide radicals (O_2_^−•^), a precursor of the various ROS. Hydroxyl radicals (OH^•^) are generated from O_2_^−•^ through a Fenton reaction. Alternatively, SOD catalyzes the formation of hydrogen peroxide (H_2_O_2_). (b) ROS are mainly generated at various complex of the respiratory chain, located in the inter-membrane space of the mitochondria. (c) ROS scavenging. The degradation of ROS is catalyzed by scavenging proteins, that is, glutathione peroxidases, catalase, and peroxiredoxin (Prx). Prx are dimers, which contain cysteine (–SH groups) (1). In the presence of H_2_O_2_, the cysteine –SH groups are oxidized to –SOH (2), which then condensed to form disulfide groups (S–S) (3). The S–S groups of Prx are reduced by thioredoxin (Trx) to return to the initial –SH form (1). PRX-SOH can be overoxidized by H_2_O_2_ to form Prx-SO_2_H groups (4).

ROS is mainly produced in mitochondria of the cells. The primary source of mitochondrial ROS is the leakage of a small fraction of respiratory chain electrons (1-2%), which react with molecular O_2_ to form superoxide ions O_2_^−•^, a precursor of various types of ROS (Figure 1(a)) [13]. The dismutation of O_2_^−•^ generates H_2_O_2_ and this reaction is catalyzed by superoxide dismutases (SOD) such as MnSOD [13]. Several mitochondrial complexes of the electron transport chain contribute to the ROS generation in MSCs and PSCs. Complex I is composed of nicotinamide adenine dinucleotide phosphate (NADPH) oxidases (NOXs) [13], the transmembrane proteins that catalyze the oxidation of NAPDH (Figure 1(b)). NOX-1 and NOX-4 are the most expressed NOX isoforms in MSCs and PSCs, and these enzymes significantly contribute to total ROS generation in the cells [14, 15]. Importantly, activation of Ras-related C3 botulinum toxin substrate 1 (Rac-1), a Rho GTPase, is required for ROS generation mediated by NOXs [16]. Complex II comprises succinate dehydrogenases, which are expressed at significant levels in undifferentiated MSCs and PSCs [17, 18]. Succinate dehydrogenase catalyzes the oxidation of succinate to fumarate. This reaction is mediated by the conversion of flavin adenine dinucleotide (FAD) to FADH2, where the intermediary electron transfer promotes ROS generation [19]. Complex III contains the ubiquinol-cytochrome c reductases, which catalyze the reduction of cytochrome c by the oxidation of coenzyme Q (Figure 1(c)). The electron leakage from the reduction of cytochrome c leads to the ROS generation [13]. Therefore, treatment of PSCs and MSCs with antimycin A (a cytochrome c reductase inhibitor) increases ROS generation [20, 21]. Complex IV is composed of cytochrome c oxidase, which mediates the oxidation of cytochrome c [13]. While the presence of complex IV has been characterized in PSCs and MSCs [22, 23], limited information is available on its contribution to ROS generation in stem cells.

The specific contribution of each mitochondrial complex to the level of ROS generation in stem cells has not been well understood and thus remains to be further explored. In addition, various metabolic intermediates of oxidative phosphorylation (e.g., 2-oxoglutarate dehydrogenase, pyruvate dehydrogenase, glycerol 3-phosphate dehydrogenase etc.) contribute differently to the level of ROS production in the specified sites of mitochondria [24]. While the ROS are mainly generated from mitochondria, other cellular compartments such as endoplasmic reticulum or lysosomes also contribute to the generation of prooxidative molecules [25]. These additional sources of ROS may also play important roles in the regulation of cellular redox status.

### 2.2. ROS Scavenging in Stem Cells

To counteract excessive accumulation of ROS, various types of scavengers are generated to regulate the redox homeostasis in stem cells, which include SODs, glutathione peroxidase (Gpx), preoxiredoxins (Prx), and lysosomal catalases (CAT) [26]. SOD enables the conversion of O_2_^•−^ to O_2_ and H_2_O_2_ by sequential oxidation-reduction of metalloproteins (i.e., Zn or Mn bound proteins) of the enzyme catalytic sites as well as the concomitant oxidation-reduction of superoxide ions [27]. Gpx enables the H_2_O_2_ reduction, in which seleno-cysteine of the enzyme serves as the substrate [28]. H_2_O_2_ is converted to H_2_O by the oxidation of N-terminal cysteine of Prxs, resulting in the formation of Prx homodimer [29]. Also, H_2_O_2_ reacts with various ferric states of the heme of the catalytic site of CAT, which enables its dismutation in a two-electron redox reaction (Figure 1(c)) [30].

The expression of cystine transporters (i.e., xCT antiporter) plays a key role in maintaining the level of antioxidant synthesis [31]. Indeed, cysteine is the major amino acid source for Gpx synthesis. However, upon oxidation in air, cysteine is converted to cystine. Facilitating the transport of cystine to cytoplasm through xCT enables the reconversion of cystine to cysteine and consequently the sustained synthesis of Gpx [31]. Moreover, under oxidative stress, the induction of nuclear factor erythroid 2-related factor 2 (Nrf-2), a regulator of the cystine transporter, leads to the increased xCT expression which results in the enhanced Gpx production [31].

Human MSCs have oxidative defense mechanism and resistance to oxidative stress. For example, MSCs express significant levels of active forms of CAT, GPx, and SOD, which confers the resistance against acute ROS-mediated apoptosis [32, 33]. Indeed, the knockdown of Gpx reduces the viability when MSCs are exposed to high concentration of H_2_O_2_ [32]. Similarly, the activities of SOD, Gpx, and CAT are significantly diminished in culture medium containing the reduced concentration of selenite [33]. The enrichment of selenite promotes Gpx and thioredoxin (Trx) activities, leading to the better resistance against oxidative stress induced by H_2_O_2_ [33]. In addition, MSCs express xCT which enables efficient cystine transport and results in the significant expression of Gpx [34, 35]. Moreover, upon acute oxidative stress, the activation of Nrf-2 leads to the increased Gpx expression [36].

Undifferentiated PSCs exhibit the increased oxidative defense compared to the differentiated cells [37]. Indeed, ESCs express a significant amount of Gpx-1, which is required to sustain their self-renewal [38]. Also, both human and mouse ESCs express large amounts of Gpx-1, SOD, Prx, and Trx, which are down-regulated during differentiation and are associated with the increased levels of intracellular ROS [37, 39]. PSCs also express a large amount of Nrf-2, which is down-regulated during spontaneous differentiation [40]. Under oxidative stress, increased Nrf-2 nuclear translocation has been observed in ESCs, which is linked to the enhanced expression of thioredoxin reductase 1, Gpx-1, and Gpx-4 [41]. However, it is suggested that undifferentiated PSCs do not express xCT [31]. As a consequence, PSCs are usually cultivated on feeder layers, which are necessary to provide a sufficient amount of cysteine for Gpx expression. Alternatively, addition of *β*-mercaptoethanol to the PSC culture medium enables the stabilization of cysteine [31].

## 3. Physiological Roles of ROS in Stem Cell Homeostasis

A tight balance between ROS generation and antioxidant protein synthesis regulates the redox homeostasis in stem cells [42]. Basal levels of ROS are required for the activation of several key cellular pathways for stem cell proliferation and survival. In the meanwhile excessive accumulation of ROS leads to cellular damage.

### 3.1. Low Levels of ROS Are Secondary Signaling Messengers for Cell Proliferation and Survival

**Figure 2:**
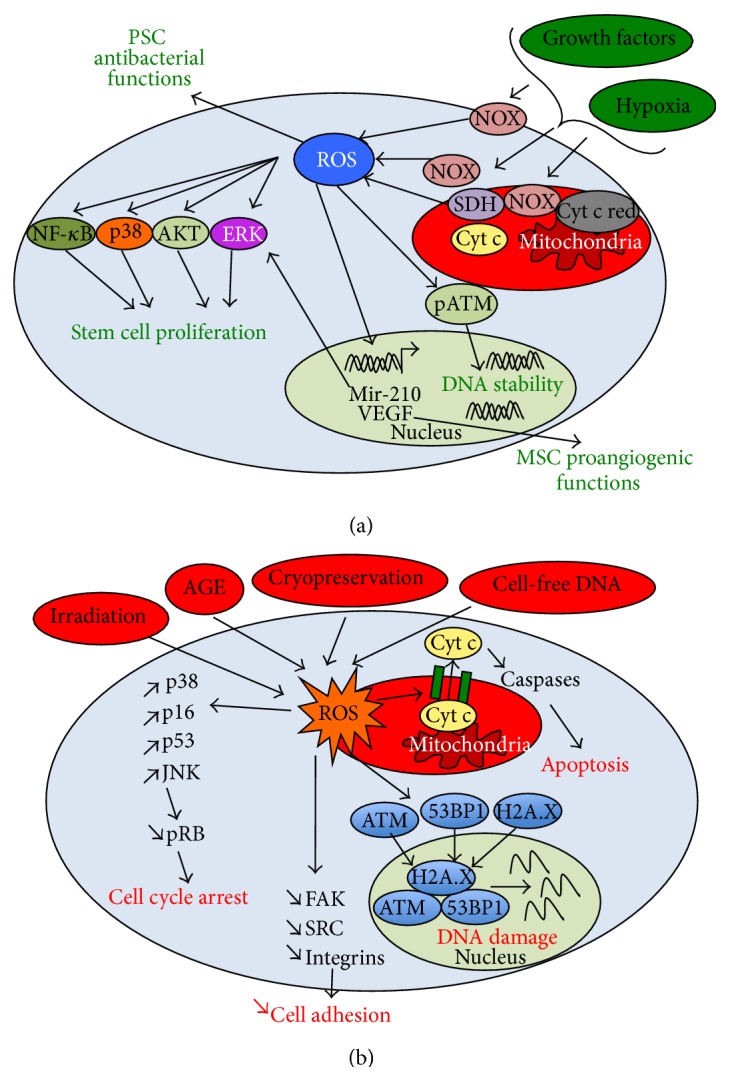
Regulation of stem cell proliferation and survival by ROS. (a) Effect of physiological levels of ROS. Physiological levels of ROS activate various MAPKs (i.e., ERK, p38, and AKT), promoting stem cell proliferation. Mir-210 is upregulated by ROS and is also involved in the MAPK activation, leading to the enhanced stem cell proliferation. Mild levels of ROS promote the activation of pATM, increasing the DNA stability of stem cells. Mild ROS can sustain antibacterial properties of PSCs and the proangiogenic functions of MSCs. (b) Effect of excessive levels of ROS. Excessive levels of ROS induce oxidative stress in stem cells. High levels of ROS result in a decreased stem cell adhesion (i.e., through a decreased activation of FAK-Src signaling) and proliferation (through the reduced activation of pRB). High ROS also reduce DNA stability and induce apoptosis (in favoring BAX-BAK dimerization and the formation of channel facilitating cytochrome c (cyt c) cytoplasmic translocation). In (a), Cyt c red means Cyt c reductase.

Low concentrations of exogenous H_2_O_2_, inhibitors of the mitochondrial electron transfer (e.g., antimycin A, rotenone), and hypoxia induce moderate levels of ROS in MSCs. Low levels of ROS are reported to enhance MSC proliferation and migration through the activation of extracellular-signal-regulated kinases (ERK) 1/2 and Jun-1/2 pathways [43–45]. The positive effects of ROS on MSC expansion are mediated by NOXs. Indeed, the knockdown of NOX-1 and NOX-4 prevents the proliferation of MSCs under hypoxia or upon cultivation in IL-7 containing medium [46]. In addition, growth factors such as platelet-derived growth factor-BB lead to the mild ROS generation which increases the proliferation and migration of adipose-derived MSCs [47]. As recently demonstrated, various mild ROS inducers (i.e., hypoxia, mitochondrial inhibitors, growth factors etc.) converge to activate miR-210, a miRNA that triggers ERK1/2 and AKT activation in MSCs (Figure 2(a)) [47]. Alternatively, moderate levels of ROS regulate the secretory function of MSCs. For instance, the induction of ROS with advanced glycation end products (AGE) is reported to promote the secretion of chemokines (e.g., CCL-2, CCL-4) by MSCs, through the activation of p38-mitogen-activated protein kinases (MAPK) pathway [48]. Also, hypoxia-induced ROS mediates pro-angiogenic function of MSCs (i.e., secretion of vascular endothelial growth factor (VEGF)) [10]. As reported for various cell types, NOXs may play a role in the trophic function through ROS generation (Figure 2(a)) [49].

Low levels of ROS generated from hypoxia also mediate the proliferation of PSCs, through the enhanced activation of the MAPK, nuclear factor-*κ*B (NF-*κ*B), and Wnt signaling (Figure 2(a)) [11, 50]. Moreover, the genetic stability of ESCs requires basal levels of ROS expression [51]. Indeed, the inhibition of ROS generation via acute dose of antioxidants (e.g., CAT) is reported to inhibit the activity of phosphorylated ataxia telangiectasia mutated (pATM), a serine/threonine protein kinase that is activated by DNA double-strand breaks. Inhibition of pATM activity is normally required for maintaining DNA integrity (Figure 2(a)) [51]. Also, the antibacterial function of ESCs is found to be controlled by the sustained expression of NOX-2 [52]. Hence, moderate levels of ROS support important physiological functions in stem cells. In contrast, the abrogation of ROS generation leads to “reductive stress” that significantly alters stem cell homeostasis [53].

### 3.2. Pathological Levels of ROS Accumulation Lead to Oxidative Stress and Cell Damage

Excessive generation of endogenous ROS and the imbalance between ROS and antioxidant proteins, as well as the culture of stem cells with various extracellular sources of ROS can lead to oxidative stress in stem cells (Figure 2(b)). For examples, aging significantly contributes to endogenously induced redox imbalance in MSCs [54]. The production of AGE, by-products of glycolysis, also leads to oxidative stress in MSCs [55]. In addition, some culture conditions such as the cryopreservation and the irradiation result in acute oxidative damages in PSCs and MSCs through endogenous ROS production [56, 57]. The presence of circulating cell-free DNA in culture medium has also been reported to induce oxidative stress in MSCs [36]. Moreover, exogenous H_2_O_2_ can diffuse through cytoplasmic membrane, leading to the oxidative stress in PSCs and MSCs. Of importance, the paracrine diffusion of endogenous ROS in MSCs has been demonstrated, indicating the propagation of oxidative imbalance at the cellular level [58].

Oxidative stress due to high levels of ROS impairs stem cell homeostasis. Indeed, high levels of ROS disturb MSC adhesion through the down-regulation of the activated focal adhesion kinase (FAK), Src, and the integrin expression (Figure 2(b)) [59]. In addition, oxidative stress leads to DNA damage in MSCs through the induction of the colocalization of ATM, H2A.X, and 53BP1 genes, the specific DNA damage response [60]. Similarly, oxidative stress reduces the telomere length of MSCs by decreasing the expression of telomeric repeat binding factor (TRF) 1 and TRF2 (two proteins involved in telomere elongation and stabilization), leading to the cell senescence (Figure 2(b)) [60, 61]. In contrast, it has been demonstrated that PSCs are less prone to DNA damage and the senescence induced by oxidative stress, due to the capability for the repair of DNA double-strand breaks [57, 62].

Oxidative stress also causes cell cycle arrest in stem cells. High ROS has been found to mediate the activation of p38-MAPK and p16, which inhibits the phosphorylated retinoblastoma (pRB) protein and causes the growth arrest of MSCs [63]. In addition, ROS has been reported to induce growth inhibition through the activation of p53 in MSCs [64]. Similarly, cell cycle arrest can be induced in mouse ESCs under oxidative stress [57, 62], as a consequence of c-Jun N-terminal kinase (JNK) and p53 activation (Figure 2(b)) [65].

Finally, excessive levels of ROS promote the apoptosis in MSCs and PSCs [62, 66]. Indeed, under oxidative stress ROS disrupts mitochondrial cardiolipin-cytochrome c complexes, liberating cytochrome c in a free form. In addition, ROS induces the BAX-BAK dimerization, enabling the formation of channels on the mitochondrial membrane and thus facilitating the translocation of cytochrome c to the cytoplasm. The cytoplasmic cytochrome c activates the expression of caspases, leading to apoptosis (Figure 2(b)) [67].

Hence, ROS play dual role in stem cell homeostasis, depending on the level of production. The exact threshold level of ROS to decipher between its role as a secondary physiological messenger or as a source of oxidative stress still needs to be further delineated.

## 4. ROS Regulates the Balance between Self-Renewal and Differentiation of Stem Cells

ROS and the oxidative defense signaling interfere with MSC and PSC differentiation pathways. The regulation of oxidant defense between undifferentiated stem cells and their differentiated progeny indicates the important role of ROS in the regulation of stem cell fate.

### 4.1. Mesenchymal Stem Cells

**Figure 3:**
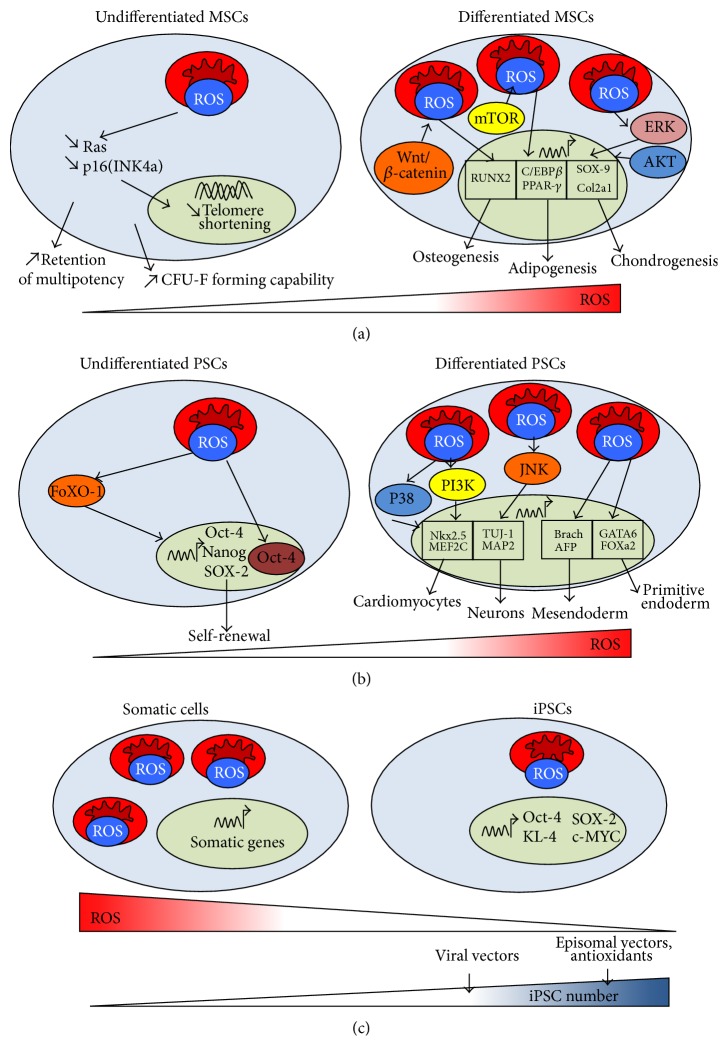
Regulation of stem cell self-renewal, differentiation, and reprogramming by ROS. (a) MSCs. MSCs have low numbers of mitochondria that generate low levels of ROS at undifferentiated state. Low ROS generation sustains the capability of forming colony-forming unit-fibroblasts (CFU-F). Upon differentiation, the number of mitochondria and the ROS levels are increased. ROS promotes the adipogenesis, osteogenesis, and chondrogenesis from MSCs. (b) PSCs. Undifferentiated PSCs have low mitochondrial biogenesis, which increases upon differentiation. Mild levels of ROS promote PSC self-renewal and the expression of Oct-4, NANOG, and SOX-2. Alternatively, the spontaneous and lineage-specific differentiations are associated with the increased ROS generation. For examples, ROS triggers PI3K and p38, which support cardiomyogenic differentiation. ROS also enhances neuronal differentiation through JNK activation. (c) iPSCs. During reprogramming, iPSCs show the decreased mitochondria biogenesis and a reduction of ROS production compared to the somatic cells. Increasing ROS scavenging during reprogramming enhances the efficiency of iPSC generation, which depends on the methods of OKSM (Oct-4, Klf-4, SOX-2, and c-MYC) transfection.

MSCs have low numbers of mitochondria at the undifferentiated state, while the increased mitochondrial biogenesis and oxidative phosphorylation (OXPHOS) supercomplexes are observed during differentiation [18, 23]. Therefore, the redox status is changed upon MSC differentiation and the spontaneous increase in ROS generation occurs during osteogenic and adipogenic differentiation (Figure 3(a)) [68]. However, MSCs display the unique redox profile depending on the differentiation path. For instance, MSCs committed to adipocyte lineage show the increased cysteine redox potential compared to the cells committed to osteoblast lineage [68].

During adipogenic differentiation of MSCs, ROS generation is increased through the mediation by NOX-4 [69, 70]. Inhibition of mitochondrial complex III and complex I significantly reduces the expression of CCAAT-enhancer-binding protein (C/EBP)-*β* and peroxisome proliferator-activated receptor (PPAR)-*γ*, the markers for adipocyte differentiation [69]. However, NOX-4 silencing (by siRNA) did not attenuate C/EBP-*β* or PPAR-*γ* expression but reduced lipid accumulation, indicating the contribution of NOX-4 at late stage of adipogenic differentiation [69, 70]. Similarly, the activation of mammalian target of rapamycin (mTOR) signaling promotes ROS generation mediated by complex III, which induces the expression of PPAR-*γ* (Figure 3(a)) [15]. To balance the increased ROS expression, adipogenic differentiation is associated with the up-regulation of Forkhead box Os (FoXOs) which regulate the expression of antioxidant enzymes (e.g. catalases, SODs, and Gpxs) [71]. However, Sirt-1 (an activator of FoXO-1) reduces adipogenic differentiation potentially by deacetylating PPAR-*γ* or impairing the formation of FoXO-1/PPAR-*γ* complexes [72, 73]. Similarly, Sirt-2 reduces adipogenic differentiation by deacetylating FoXO-1, which promotes nuclear localization of FoXO-1 [74].

During osteogenic differentiation of MSCs, canonical Wnt/*β*-catenin induces ROS generation and plays a critical role in the regulation of MSC differentiation by activating osteogenesis and inhibiting adipogenesis [75]. To balance the ROS effects, the activation of FoXO-1 promotes osteogenic differentiation by regulating the expression of RUNX-2, a master regulatory factor of osteogenesis [76]. Moreover, Sirt-1 enables the deacetylation of *β*-catenin and promotes nuclear accumulation of *β*-catenin, which acts as a transcription factor of osteogenic genes [77]. The imbalance between the level of ROS and the expression of scavenging proteins can lead to the reduced osteogenic differentiation of MSCs (Figure 3(a)) [78].

ROS generation is also increased during chondrogenic differentiation [79]. ROS produced through NOX-2 and NOX-4 promotes cell survival during chondrogenesis [79]. Moreover, endogenous ROS triggers AKT and ERK signaling, leading to the enhanced expression of SOX-9, collagen type II and the accumulation of proteoglycans (Figure 3(a)) [79]. Sirt-1 is required for chondrogenic differentiation of MSCs through the activation of SOX-9 as well as the deacetylation of NF-*κ*B, leading to the decreased expression of matrix metalloproteinase (MMP)-9, COX-2, and caspase-3 [80]. Finally, the FoXOs enhance the survival of MSCs and prevent the differentiation towards hypertrophic chondrocytes (i.e., indicated by the expression of collagen type X) [81].

### 4.2. Pluripotent Stem Cells

PSCs display low levels of ROS expression at the undifferentiated state as a consequence of a low level of mitochondrial biogenesis and a high level of ROS scavenging protein expression (e.g. Gpx-1) [38, 82]. The basal levels of ROS are required to sustain the self-renewal of PSCs [17, 83]. Indeed, it is recently demonstrated that ROS modulates Oct-4 posttranslational modifications (such as sumoylation and ubiquitination), leading to the enhanced nuclear localization of Oct-4 [84]. Sirt-1, a key cell survival factor activated upon ROS exposure, is down-regulated during ROS-induced differentiation through the activity of miR-29b [85]. Sirt-1 also regulates the activation of FoXOs which are required to maintain pluripotency by directly regulating the expression levels of Oct-4, Nanog, and SOX-2 in human ESCs (Figure 3(b)) [86, 87].

The redox status of PSCs changes significantly during spontaneous differentiation. Indeed, ROS expression is increased upon PSC lineage commitment as a consequence of the regulation of scavenging protein expression. For instance, the expressions of Prx-1, SOD2, Gpxs and CAT are found lower in embryoid bodies compared to undifferentiated ESCs [37, 88]. ROS generation by retinoic acid enables the activation of Wnt signaling through the increased expression of NOXs during extra-embryonic endodermal differentiation (i.e., indicated by the expressions of GATA-6 and FOXa2) of PSCs [89, 90]. In addition, ROS generation through the inhibition of glutathione mediates the differentiation of human ESCs towards mesodermal (i.e., indicated by the expression of brachyury, myogenin, and myogenic factor 6) and endodermal lineages (i.e., indicated by the expression of HNF3*β*, AFP, and Sox17) [91]. ROS is found to regulate meso-endodermal specification through the modulation of the MAPKs, such as the inactivation of p38 and AKT as well as concomitant transient increase of JNK and ERK signaling (Figure 3(b)) [91].

ROS also mediates the lineage-specific differentiation of PSCs. For instance, the icariin-induced ROS production through NOX-4 promotes ESC differentiation into cardiomyocytes [92]. ROS can trigger p38 activation and phosphatidylinositol-4,5-bisphosphate 3-kinase (PI3K) expression, which mediate MEF2C nuclear translocation, a key transcription factor in ESC cardiac differentiation [93]. ROS produced during cardiac differentiation is also found to activate NF-*κ*B signaling and trigger phosphatidylinositol 3-kinase enhancer (PIKE) and PI3K activation [94]. Similarly, ESC differentiation into smooth muscle cells shows the increased ROS production, which mediates the nuclear translocation of serum response factor (SRF), a transcription factor specific for smooth muscle cells [95]. Neural differentiation of ESCs also demonstrates the increased ROS generation through the regulation of antioxidant protein expression [88]. Indeed, it has been recently demonstrated that the Prxs knock-down in mouse ESCs prevents ROS-mediated activation of JNK signaling, which is required for neuronal differentiation (Figure 3(b)) [88].

### 4.3. Influence of ROS during iPSC Reprogramming

Recently, the reprogramming of somatic cells through forced expression of a set of genes (Oct-4, KL-4, SOX-2, and c-MYC, i.e., OKSM) enables the generation of iPSCs that display ESC-like properties [4]. Both iPSCs and ESCs demonstrate low redox status and the capability for DNA repair following oxidative damage [96]. Importantly, the somatic mitochondria can revert to an ESC-like state in terms of morphology, cellular distribution, and rate of biogenesis after reprogramming (Figure 3(c)) [97].

However, somatic cell reprogramming to derive iPSCs through viral transfection is associated with a high level of ROS which leads to oxidative damage [98, 99]. The oxidative stress generated during reprogramming impairs cell survival and promotes genetic aberrations [98, 99]. Addition of antioxidants (such as N-acetylcysteine or vitamin C) improves the reprogramming efficiency and reduces genetic abnormalities [98, 99]. Among OKSM genes, c-MYC is reported to be involved in a high level of ROS generation during iPSC derivation [98]. In addition, various methods of reprogramming lead to different levels of ROS generation. For instance, the episomal transfection of OKSM generates lower amounts of ROS than the viral-based reprogramming [100].

Together, ROS play an important role in the regulation of stem cell self-renewal, differentiation and reprogramming. However, the threshold levels for ROS and the regulation of scavenging protein expression to modulate stem cell fates still need to be defined.

## 5. Modulation of ROS Generation through Stem Cell Microenvironment

**Figure 4:**
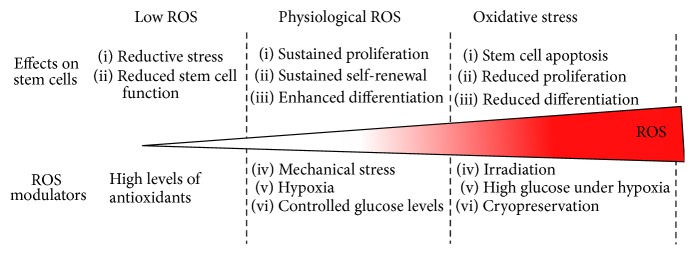
Modulation of ROS level regulates the stem cell fate decision. ROS play dual role in stem cell homeostasis, depending on its level of production. At mild levels, ROS serves as a secondary physiological messenger. At high levels, ROS is a source of oxidative stress that leads to cell apoptosis. Tight regulation of biochemical and biomechanical environment can control stem cell oxidative status and thus the stem cell fate decision.

Stem cell microenvironment including cellular organization and physiological parameters is a potent regulator of ROS generation. Modulating stem cell microenvironment could lead to a better control of redox status for stem cell proliferation and differentiation. The contribution of the respiratory chain in ROS generation suggests the intricate link between stem cell metabolism and ROS generation [101]. In addition, the pathway involving Rac-1 is activated in MSCs and PSCs upon biomechanical stimulation, indicating the relation of mechano-transduction with ROS generation. Thus, tight regulation of biochemical and biomechanical environment can control stem cell oxidative status towards efficient expansion and differentiation (Figure 4).

### 5.1. Biochemical and Metabolic Regulation of ROS Generation

The metabolism of undifferentiated MSCs and PSCs mainly relies on glycolysis, while a metabolic shift towards OXPHOS is generally observed upon differentiation [12]. The reverse process is also demonstrated during reprogramming where the metabolism of somatic cells shifts from OXPHOS to glycolysis when gaining pluripotency [12]. OXPHOS mediates the production of ROS as a consequence of electron leakage from the respiratory chain. Due to the dominant glycolytic metabolism, undifferentiated MSCs and PSCs generate lower levels of ROS than their differentiated counterparts [101].

Due to the involvement of glucose in metabolic pathways, glucose concentration in the culture medium affects ROS generation in stem cells, through the regulation in the expression of mitochondrial complexes [102]. As a consequence, ROS production is increased when MSCs and PSCs are cultivated in the medium containing a high level of glucose, while the low-glucose medium attenuates the production of ROS and induces antioxidant secretion in MSCs (e.g., MnSOD or catalase) [102, 103].

Oxygen tension within the stem cell microenvironment also regulates ROS generation. Low oxygen tension (i.e., hypoxia) favors glycolysis, leading to the reduced ROS production through the metabolism of MSCs and PSCs [104, 105]. However, a rapid transient increase of physiological ROS has been observed under hypoxia. The hypoxia-generated ROS is mediated through the complex III and NOXs which enable the activation of MAPK and the stabilization of hypoxia inducible factors (HIF) to regulate the survival and proliferation of MSCs and PSCs [106, 107]. In addition, hypoxia increases the reprogramming efficiency of somatic cells, potentially due to the reduced oxidative stress and the promotion of glycolysis through the stabilization of HIF-1*α* [108].

Interactions between the effects of glucose concentration and oxygen tension are observed for ROS generation in stem cells. Indeed, while low glucose condition reduces the ROS production, high glucose condition in combination with hypoxia induces oxidative stress, impairing the stem cell survival and function such as the secretion of proangiogenic factors [8]. It has been found that high glucose under hypoxia promotes the degradation of HIF-1*α* through the increased proteasome activity [8]. All these observations indicate that controlling stem cell metabolic environment can modulate ROS generation.

### 5.2. Biomechanical Signals Regulate ROS Generation

Rac-1 is a member of Rho-GTPases and is involved in cell-cell and cell-matrix adhesion, the cytoplasmic membrane ruffling, and lamelipodia elongation [109, 110]. Rac-1 is found to play a key role in the generation of ROS. Indeed, the enzymatic activity of NOXs (i.e., the conversion of NADPH to NADP) is mediated by the interactions of the enzyme's several subunits (p22phox, p47phox, 40phox, and p67phox) and Rac-1 [111]. Consequently, Rac-1 plays a key role in the regulation of PSC and MSC proliferation, migration and differentiation [112–114].

The biomechanical environment of PSCs and MSCs (such as surface pattern or stiffness) can modulate Rac-1 activation [113, 115, 116], which in turn mediates ROS generation. For example, the application of cyclic strains to mouse ESCs promotes ROS generation, which induces cardiomyogenic differentiation [14, 117]. A gradual increase in ROS generation and a concomitant decrease in SOD expression have been observed when increasing the magnitude of cyclic strain that is applied to MSCs (6 to 24% magnitude) [118]. The biomechanical regulation of ROS generation in stem cells is an emerging area and needs further exploration.

### 5.3. Extracellular Matrix and Cell Aggregation as the Modulators of Redox Status

Extracellular matrix (ECM) may bear antioxidant properties that protect stem cells from oxidative damages [119, 120]. For instance, it has been demonstrated that young endogenous ECM derived from MSCs reduces ROS production compared to old ECM or plastic dishes [119]. The endogenous glycosaminoglycans such as chondroitin sulfate or small leucine-rich proteoglycans have antioxidant properties, which may mediate stem cell oxidative defense [119, 121]. Alternatively, decellularized ECM from MSCs has been shown to promote the resistance against oxidative stress through the secretion of endogenous antioxidants such as SOD2 [122]. It has been demonstrated that ECM proteins are the targets of ROS through the activation of MMPs, which can affect MSC motility [123].

The formation of MSC aggregates promotes the secretion of ROS scavenging proteins such as SOD2, leading to the increased resistance of MSCs against acute H_2_O_2_ exposure [124, 125]. The increased secretion of antioxidant proteins might be due to mild hypoxia found in the core of MSC aggregates [124]. Similarly, PSC aggregates can display the increased anti-oxidative defense upon acute ROS generation (e.g., after cryopreservation) [56]. However, the molecular mechanism conferring oxidative defense in stem cell aggregates is still unclear.

## 6. Conclusions

While high levels of ROS have detrimental effects on stem cells through the induction of oxidative stress, physiological levels of ROS play an important role in the regulation of stem cell fate decision. Mild levels of ROS act as secondary messengers by interfering with various signaling pathways that regulate stem cell proliferation, survival, and differentiation. However, the contribution of the specific site of ROS production and the specific type of ROS in the regulation of stem cell fate requires further delineation. In addition, the exact threshold levels of ROS deciphering between the role as damaging molecules or as enhancers of stem cell signaling pathway are not clearly defined. Therefore, methods for the in situ detection of ROS level and the specific species are required to accurately quantify and characterize the threshold level of ROS to modulate stem cell homeostasis. For instance, the application of Raman spectrometry or alternative probes may be preferred to measure intracellular ROS [126]. Combined with accurate ROS measurement, regulation of biochemical and biomechanical environment of stem cells to modulate redox status can lead to the controlled proliferation and differentiation of stem cells towards various biomedical applications.

## Abbreviations

AGE: Advanced glycation end products
CAT: Lysosomal catalase
C/EBP: CCAAT-enhancer-binding protein
CFU-F: Colony-forming unit-fibroblasts
ECM: Extracellular matrix
ESC: Embryonic stem cell
ERK: Extracellular-signal-regulated kinase
FAD: Flavin adenine dinucleotide
FAK: Focal adhesion kinase
FoXOs: Forkhead box Os
Gpx: Glutathione peroxidase
iPSC: Induced pluripotent stem cell
JNK: c-Jun N-terminal kinase
HIF: Hypoxia inducible factor
MAPK: Mitogen-activated protein kinase
MMP: Matrix metalloproteinase
MSC: Mesenchymal stem cell
mTOR: Mammalian target of rapamycin
NADPH: Nicotinamide adenine dinucleotide phosphate
NOXs: NADPH oxidases
NF-*κ*B: Nuclear factor-*κ*B
Nrf-2: Nuclear factor erythroid 2-related factor 2
OXPHOS: Oxidative phosphorylation
pATM: Phosphorylated ataxia telangiectasia mutated
pRB: Phosphorylated retinoblastoma protein
PI3K: Phosphatidylinositol-4,5-bisphosphate 3-kinase
PIKE: Phosphatidylinositol 3-kinase enhancer
PPAR: Peroxisome proliferator-activated receptor
Prx: Preoxiredoxin
PSC: Pluripotent stem cell
Rac-1: Ras-related C3 botulinum toxin substrate 1
ROS: Reactive oxygen species
SOD: Superoxide dismutase
SRF: Serum response factor
TRF: Telomeric repeat binding factor
Trx: Thioredoxin
VEGF: Vascular endothelial growth factor.
